# Antiproliferative Activity and Antioxidant Potential of Extracts of *Garcinia gardneriana*

**DOI:** 10.3390/molecules25143201

**Published:** 2020-07-14

**Authors:** Simone da Cunha Demenciano, Magalli Costa Barbosa Lima e Silva, Caroline Almeida Farias Alexandrino, Wilson Hino Kato Junior, Patrícia de Oliveira Figueiredo, Walmir Silva Garcez, Raquel Pires Campos, Rita de Cássia Avellaneda Guimarães, Ulana Chaves Sarmento, Danielle Bogo

**Affiliations:** 1 Molecular Biology and Cell Culture Laboratory, School of Pharmaceutical Sciences, Foods and Nutrition (Facfan), Federal University of Mato Grosso do Sul (UFMS), Cidade Universitária, Campo Grande 79070-900, Brazil; sinutri_2@hotmail.com (S.d.C.D.); magallicbls@gmail.com (M.C.B.L.eS.); carolfalexandrino@gmail.com (C.A.F.A.); danielle.bogo@ufms.br (D.B.); 2 Graduate Program in Health and Development in the Central-West Region of Brazil, Federal University of Mato Grosso do Sul, Campo Grande 79079-900, Brazil; walmirgarcez@gmail.com; 3 Laboratory PRONABio (Laboratory of Bioactive Natural Products)—Chemistry Institute, Federal University of Mato Grosso do Sul, Cidade Universitária, Campo Grande 79070-900, Brazil; hinokato@gmail.com (W.H.K.J.); patricia.figueiredo@ufms.br (P.d.O.F.); raquel.campos@ufms.br (R.P.C.); 4 Laboratory of Physical Chemistry of Foods, School of Pharmaceutical Sciences, Foods, Federal University of Mato Grosso do Sul (UFMS), Cidade Universitária, Campo Grande 79070-900, Brazil; ulanachaves@hotmail.com

**Keywords:** bacupari, anticancer, HPLC-DAD-MS, brazilian cerrado fruits, cytotoxicity

## Abstract

The aim of this study was to evaluate the antiproliferative activity, the antioxidant potential, and the chemical profile obtained from the whole fruit and from leaves of *Garcinia gardneriana*, a fruit tree from Brazilian Cerrado. To determine in vitro antiproliferative activity, the following neoplastic cell lines were considered, along with an immortalized nontumor cell line. The antioxidant potential was determined, and the evaluation of antiradical air activity was performed. The levels of vitamin C and carotenoids were determined. The chemical profile was analyzed by high-performance liquid chromatography coupled to a diode array detector and a mass spectrometer using electrospray ionization interface. The chloroform fraction of the leaf showed antioxidant activity. The vitamin C content had lower values in fruits and higher in leaves. The content of carotenoids for fruits and leaves was expressive. The ethanolic extract and the hexane and chloroform fractions of fruits were active in all neoplastic lines tested. The leaves showed cytotoxic activity in the hexane fraction in the breast carcinoma line. The analysis of data obtained verified the presence of dimers, monomers, and tetramers of hexoses, polycarboxylic acids, xanthones, flavonoids, biflavonoids, and benzophenones.

## 1. Introduction

The Brazilian Cerrado is the second largest biome in Brazil, occupying 24% of the national territory. The vegetation of this biome is characterized by a diversity of native fruits with peculiar sensory characteristics and high nutritional and economic potential. Fruit consumption has grown in the national and international markets as a result of access to health information [1].

Known as a natural source of bioactive compounds, there is great interest in preserving and valuing local biodiversity to encourage consumption and use of Brazilian Cerrado’s fruits. They adapt to the soils of the region with low implementation costs and show great species richness, which allows them to be classified as “plants of the future” [2].

Phytochemical studies have revealed the presence of several bioactive secondary metabolites for the species and genus *Garcinia*, such as phenolic compounds, mainly xanthones [3,4,5,6,7], and benzophenones and bioflavonoids [8,9,10,11]. Among these species is *Garcinia gardneriana* (Planchon & Triana) Zappi, popularly known as bacupari, which has been used in folk medicine to treat inflammation, pain, urinary infections, and other infections. *Garcinia brasiliensis* shows important antimicrobial and antioxidant properties in leaves, making its use a potential for the development of new drugs to treat cutaneous lesions [12].

Phenolic substances are efficient natural antioxidants, avoiding oxidative stress. The oxidative process may be the etiology of several chronic noncommunicable diseases, such as atherosclerosis, diabetes, obesity, neurodegenerative disorders, and cancer [13]. Cases involving high levels of oxidative stress or DNA damage have been reported in human malignancies [14].

Cancer is one of the chronic diseases with the highest mortality rate in the world. Its origin is fundamentally genetic and developed by the accumulation of mutations in cellular DNA. Physical and chemical mutagenic agents in the environment or toxic products of the cell itself, such as free radicals, can also contribute to its development. Thus, substances with an antioxidant activity act against oxidative stress, preventing such diseases [15].

In addition, there are studies on the anticancer activity of plants belonging to the genus *Garcinia*, such as the case of *Garcinia dulcis* fruit methanolic extract, which has anticancer activity in the liver cancer cell line (HepG2) [16,17].

The isolation of compounds is essential for the development of new substances with antiproliferative and antioxidant activities [18]. Thus, considering Garcinia species was proven to be a rich source of active metabolites, including reports of antioxidant and anticancer activities, this study determines the chemical composition of *G. gardneriana* fruits and leaves and evaluates the antiproliferative activity against a panel of cancer cell lines and the free radical scavenging activity of the respective extracts and fractions in order to clarify whether this plant is a potential source of prototypes to drugs with anticancer activity.

## 2. Results

### 2.1. Identification of the Constituents by HPLC-DAD (Diode Array Detector)-MS

**Figure 1:**
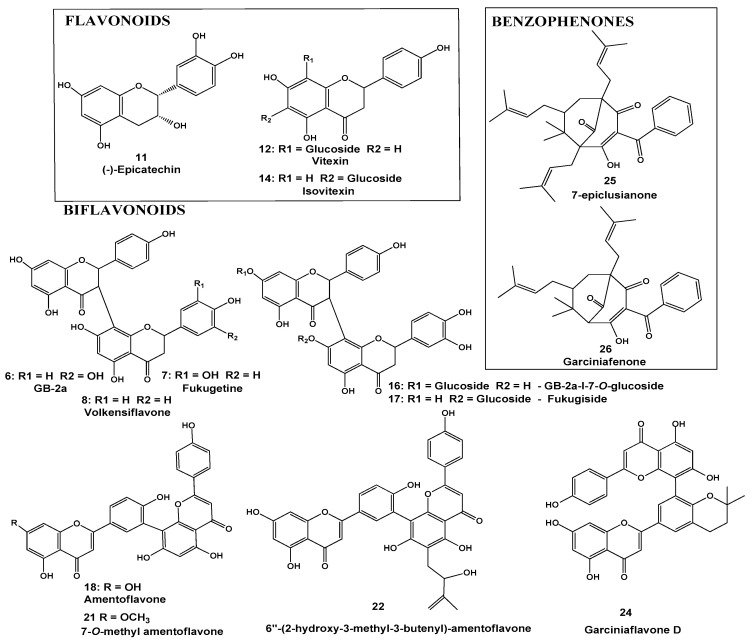
Compounds identified in the ethanol extracts of fruits (EEF) and leaves (EEL) of *Garcinia gardneriana*.

The determination of the chemical profiles of FEE (fruit ethanolic extract), EEFH (hexane fraction), EEFC (chloroform fraction), EEFA (ethyl acetate fraction), EEFW (hydromethanolic fraction), EEL (ethanol leaf extract), EELH (hexane fraction), EELC (chloroform fraction), EELA (ethyl acetate fraction), and EELW (hydromethanolic fraction) were performed based on UV, MS, and MS/MS data as compared with data described in the literature [11,12,13,14,15,16,17,18,19,20,21,22,23,24,25]. Twenty-six compounds were tentatively identified (Table 1 and Table 2, Figure 1). They are monomers, dimers, and tetramers of hexoses (**1**, **2**, and **9**), polycarboxylic acids (**3**–**5**), xanthones (**15**, **11**, **19**, and **22**), flavonoids (**11**–**14**), biflavonoids (**6**–**8**, **10**, **16**–**18**, **20**, **21**, and **23**), and benzophenones (**24** and **25**) (Figure 1).

The HPLC-DAD-MS analysis of EEF (ethanol fruits’ extract) and fractions resulted in the identification of the compounds listed in Table 1.

Among the compounds identified, the presence of citric acid lactone (**3**) stands out. Biflavonoids GB-2a (**6**), fukugetine (**7**), and volkensiflavone (**8**) were also identified.

**Figure 2:**
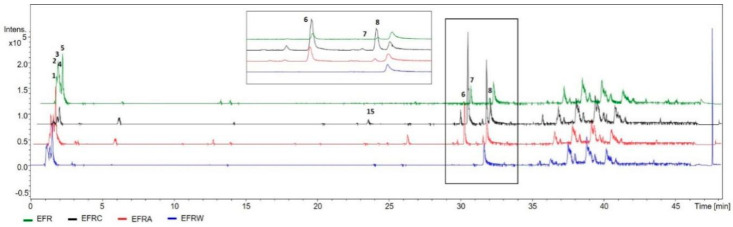
Base peak chromatogram (BPC) obtained by HPLC-DAD-MS of EEF extract and the fractions EEFC (chloroform fraction), EEFA EEFA (ethyl acetate fraction), and EEFW (hydromethanolic fraction). The identification of chromatographic peaks is described in Table 1 and all the chromatograms are in the same intensity range.

The compounds identified in the ethanolic extract EEF were also observed in the analyzed phases EEFC, EEFA, and EEFW at different proportions (Figure 2), except for the EEFW, in which biflavonoids (**6**–**8**) were not observed. The EEFC fraction concentrated biflavonoids (**6** and **8**) and xanthone (**15**), while the EEFA fraction concentrated biflavonoid (**6**) (Figure 2).

The HPLC-DAD-MS analysis of EEL (leaf extract) resulted in the identification of the compounds listed in Table 2.

**Figure 3:**
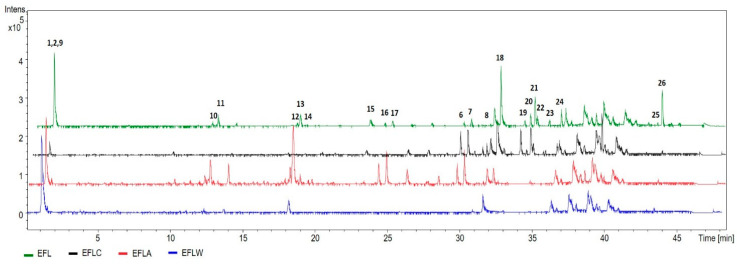
Base peak chromatogram (BPC) obtained by HPLC-DAD-MS of EEL extract and the fractions EELC (chloroform fraction), EELA (ethyl acetate fraction), and EELW (hydromethanolic fraction). The identification of chromatographic peaks is described in Table 2 and all the chromatograms are in the same intensity range.

In addition to hydroxycitric acid lactone (**3**), this extract showed xanthones (**15**, **11**, **19**, and **22**), flavonoids (**11**–**14**), biflavonoids (**6**–**8**, **10**, **16**–**18**, **20**, **21,** and **23**), and benzophenones (**24** and **25**) (Table 2). Comparing the composition of the EEL extract and its fractions, the prenylatedxanthones**15**, **11**, **19**, and **22** and the biflavonoids **20**, **21,** and **23** were found only in the chloroform fraction EELC, while the flavonoids **11**–**14** were concentrated at the EELA ethyl acetate fraction. None of the analyzed fractions (EELC, EELA, and EELW) showed the benzophenones **24** and **25**, implying that they were separated to the hexane fraction EELH (Figure 3).

### 2.2. Centesimal Composition and Antioxidant Activity of Fruits and Leaves of Garcinia gardneriana

Table 3 shows the moisture, ash, protein, lipid, and carbohydrate content of the species under study.

In leaves, a high percentage of ash, about 10 times higher than that found in fruits, indicates a high mineral content. The lipid content of fruits was 2.6 times higher than that of leaves, while the carbohydrate content of leaves was five times higher than that of fruits.

The concentration of total phenols in fruitsgallic acid equivalent (GAE), (107.07 mg GAE 100 g^−1^) was lower than that found in leaves (132.46 mg GAE 100 g^−1^) [1], classifying the content of polyphenols in fruits into three categories: Low (<100 mg GAE 100 g^−1^), moderate (100–500 mg GAE 100 g^−1^), and high (>500 mg GAE 100 g^−1^). Other studies have used this classification [26]. According to this classification, both fruits and leaves presented moderate levels of polyphenols (Table 3).

The ascorbic acid content showed lower values in *Garcinia gardneriana* fruits (25.23 mg/100 g^−1^) in comparison with leaves (30.26 mg/100 g^−1^). Vitamin C is an important natural nutrient in daily diet [27]. Fruits offer several health benefits and are part of a group of foods rich in bioactive compounds, mainly antioxidants [28]. The ascorbic acid reduces tocopherol radicals back to their active forms on cell membranes, providing anticancer potential [29].

The carotenoid contents were verified with smaller amounts in fruits (27.05 mg of β-carotene/100 g^−1^) of *Garcinia gardneriana* in relation to leaves (76.25 mg of β-carotene/100 g^−1^). Carotenoids are present mainly in vegetables and fruits and are efficient in organic protection against carcinogenesis. In vitro and in vivo tests with carotenoids have played important roles in the treatment and prevention of cancer, demonstrating an effective antioxidant action [30].

The determination of the free radical scavenging activity by the DPPH (radical scavenging activity using DPPH (1,1-diphenyl-2-picrylhydrazyl) assay) method in extracts and fractions of *G. gardneriana* fruits and leaves showed that extracts and fractions of leaves showed better activities when compared with extracts and fractions of fruits, except for the hexane fraction (Table 4).

The chloroform fraction of the leaf showed a greater antioxidant activity, that is, a greater antioxidant action than DPPH (radical estável 2,2-difenil-1-picril hidrazil), with a value ofCI_50_ 14.27 μg/mL (50% inhibition values), as well as ethyl acetate and ethanol extract fractions with values CI_50_ 16.68 μg/mL and 16.95 μg/mL, respectively. Studies carried out on the hydroalcoholic extract of bacupari leaves obtained CI_50_ of 34.83 μg/mL. So far, no reports of whole fruits have been found, but only specific and isolated analyses such as skin, pulps, and seeds [31]. In fruits, the hexane extract showed the lowest CI_50_ 20.05 μg/mL compared to the other studied fractions. The antioxidant activity in other species of *Garcinia* and verified a CI_50_ 54 μg/mL for *Garcinia humilis* and 40.77 μg/mL for *Garcinia xantochymus* [26].

### 2.3. Evaluation of the Antiproliferative Activity of Extracts and Fractions of Garcinia gardneriana Fruits and Leaves in Cancer Cell Lines

**Figure 4:**
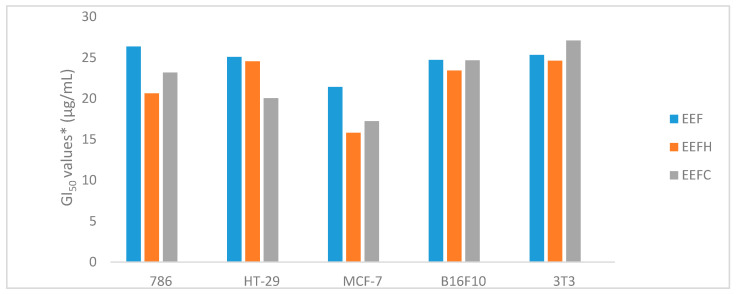
Cytotoxic activity, GI_50_ values* (μg/mL) for extracts and fractions of fruits of *Garcinia gardneriana*, lines 786 (kidney carcinoma), HT-29 (colon carcinoma), MCF-7 (breast carcinoma), B16-F10 (murine melanoma), and NIH/3T3 (murine fibroblast). * Concentration that inhibits 50% of cell growth determined by nonlinear regression analysis using the software ORIGIN 6.0. Mean value ± standard deviation, *n* = 3. *EEF* (fruit ethanolic extract), EEFH (hexane fraction), and *EEFC* (chloroform fraction).

**Figure 5:**
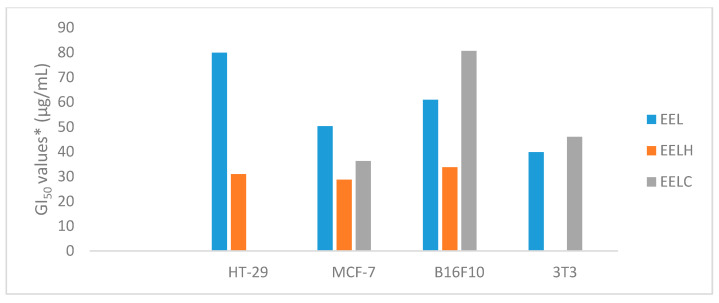
Cytotoxic activity, GI_50_ values* (μg/mL) for extracts and fractions of leaves of *Garcinia gardneriana*, HT-29 (colon carcinoma), MCF-7 (breast carcinoma), B16-F10 (murine melanoma), and NIH/3T3 (murine fibroblast). *Concentration that inhibits 50% of cell growth determined by nonlinear regression analysis using the software ORIGIN 6.0. Mean value ± standard deviation, n = 3. *EEL* (ethanol leaf extract), *EELH* (hexane fraction), and *EELC* (Chloroform fraction).

The Figure 4 and Figure 5 show values to GI_50_ (concentrations that inhibits ion 50% of cell proliferation) expressed as μg/mL, GI_50_ < 250 μg/mL, and Table 5 shows complete data. According to the National Cancer Institute (NCI), an extract that has a GI_50_ < 30 μg/mL is considered active [32].

Among the results obtained by the EEF extracts analyzed at concentrations of 0.25, 2.5, 25, and 250 µg/mL after 48 h of incubation (Figure 4), the EEF and EEFH and EEFC fractions were active in all lines, with GI_50_ ranging from 15.81 µg/mL to 27.10 µg/mL. The EEFA and EEFW fractions were not active in any of the neoplastic lines tested (GI_50_ > 250 μg/mL).

The EEL showed no cytotoxic activity in the lines tested (GI_50_ < 30 μg/mL), except for the EELH fraction, which was active in the MCF-7 line (GI_50_ = 28.7 μg/mL). The cytotoxic activity of EEF and EELC in the normal cell (NIH/3T3, murine fibroblast) was lower than that in the same extracts obtained from fruits. The EEFH was more cytotoxic (GI_50_ < 24.63 μg/mL) in fruits than in leaves. The EELA and EELHM did not show this activity (GI_50_ > 250 μg/mL).

Among the results obtained by the EEF and EEL extracts analyzed at concentrations of 0.25, 2.5, 25, and 250 µg/mL after 48 h of incubation (Table 5), EEF, EEFH, and EEFC were active against all cell lines, with GI_50_ ranging from 15.81 µg/mL to 27.10 µg/mL. The EEFA and EEFW fractions were not active in any of the cancer cell lines tested (GI_50_ > 250 μg/mL). For leaves’ extract and fractions, only the hexane fraction, EELH, showed active against MCF-7 cell line (GI_50_ = 28.7 μg/mL). The cytotoxic activity of EEF and EELC in the 3T3 line was lower than in the same extracts obtained from fruits. The EEFH was more cytotoxic (GI_50_ < 24.63 μg/mL) in fruits than in leaves. The EEFA, EEFHM, EELA, and EELHM did not show this activity (GI_50_ > 250 μg/mL).

## 3. Discussion

The chemical profile of the ethanol extracts and fractions from the fruits and leaves of *G. gardneriana*, obtained by HPLC-DAD-EM analysis, revealed the presence of a great variety of phenolic compounds, especially in leaves. The observed results, showing the presence in both fruits and leaves of flavonoids, biflavonoids, catechins, xanthones, and others, corroborate the importance of phenolic compounds as promising active components. For several of the compounds found in the extracts of fruits and leaves, studies have attributed the following bioactive potentials: Antioxidant [33,34,35], antiproliferative, and apoptotic [36,37].

Several studies carried out on plants used in traditional medicine, evidencing the presence of xanthones and flavonoids, have shown that these compounds contribute to antioxidant activities [38,39] and that catechins have antioxidant properties similar to vitamin C (ascorbic acid) that help to inhibit the action of free radicals, protecting the organism from some diseases, such as cancer [40,41].

It has already been reported as one of the main component acids of *Garcinia indica* species [18], in addition to isomers of citric acid (5, 5′) and glycosylated citric acid (4, 4′, 4″), all reported for the first time in fruits of *G. gardneriana*.

Among the phenolic compounds, the presence of flavonoids and biflavonoids identified in this chemical study may be related to the antiproliferative activity of leaf and fruit extracts. These compounds are electron donors. In their chemical structure, they present several hydroxyls that have an antioxidant action because they react and inactivate superoxide anion, singlet oxygen, and peroxide radicals and stabilize free radicals through hydrogenation or complexation with oxidizing species. They also act by inhibiting the cell cycle and inducing apoptosis [42].

The results found in this study corroborate other studies that have suggested that the consumption of exotic fruits should be stimulated as they are sources of steroidal compounds, fatty acids, pigments, and phenolic compounds that have a potential biological activity, specifically an antimutagenic activity, which is closely related with antitumor action since every tumor begins after the mutation of a normal cell [43].

The literature review of some compounds identified in *Garcinia gardneriana* extracts and fractions (Table 6) revealed they have promising activities, such as analgesic, antibacterial, antioxidant, antiviral, anti-inflammatory, antimicrobial, antifungal, cytotoxic, anti-caries, anti-parasitic, trypanocide, antiproliferative, and anti-tumor activities.

Cytotoxicity screening models provide important preliminary data to help to select plant extracts with potential antitumor properties for future bio-related studies [27]. The American National Cancer Institute guidelines assign a significant cytotoxicity effect to a promising anticancer product if the extracts have GI_50_ values lower than 30 μg/mL as well as small molecules, such as plant secondary metabolites, with a 30–50% activity cutoff and activity concentrations ranging between 1 and 50 µM (micromolar)with a potential as hit compounds [28,29].

The best GI_50_ value presented for fruits was against MCF-7 cell line (GI_50_ 15.81 μg/mL). To date, no reports have been found in the literature on the evaluation of the cytotoxicity of whole fruits (peel, pulp, and seed), as performed in this study. However, a cytotoxic study of the pericarp of *Garcinia mongostana* considering ethanolic extract and using the MTT (3-(4,5-dimethylthiazol, 2-yl)- 2,5-diphenyl-212 tetrazolium bromide) method was performed [16]. The results of the study showed an antiproliferative effect on cells of the MCF-7 human breast line (CI_50_ of 45 µg/mL). The MCF-7 line is sensitive to treatment and has an antiproliferative effect for an isolated benzophenone compound (7-*epi*-clusianone) from *G. brasiliense*, presenting a CI_50_ value of 6 µg/mL [16].

According to the data shown in the manuscript of the 10 samples evaluated for cytotoxicity in the NHI/3T3 line (murine fibroblate) nontumoral cells, five of them were not cytotoxic because their GI_50_ values were greater than 250 µg/mL. However, for the EEF, EFH, EFC, EEL, and EELC samples, the GI_50_ values were less than 50 µg/mL, thus indicating cytotoxicity.

The aqueous extract of leaves of *G. brasiliensis* in the human cancer cell line MCF-7 showed that cell viability was affected only when it was used at concentrations above 1000 μg/mL with a CI_50_ of 312.0 μg/mL ± 16.45 μg/mL [52].

In cytotoxicity tests carried out on leaves of *G. gardneriana*, the hydroalcoholic fraction (GI_50_ 16.97 μg/mL) was effective in the HL-60 line (human promyelocytic leukemia) [53,54]. In the HCT-8 line (colon, human cell line), a GI_50_ of 15.30 μg/mL was reported in the ethanolic fraction and a GI_50_ of 11.68 μg/mL was reported in the hydroalcoholic fraction. The other extracts, such as hexane and acetate, had GI_50_ > 30 μg/mL.

The presence of xanthones, benzophenones, and bioflavonoids causes an anticancer activity in several neoplastic lines even at low concentrations [54]. The xanthones already identified for the genus *Garcinia* have anti-inflammatory, antiviral (herpes), antimicrobial, antifungal, cytotoxic, and antioxidant activities, as reported [34,38,44,45]. Among them, epicatechin has antioxidant properties [34,40,41].

We can attribute the cytotoxic effects found in fruits and leaves to the presence of amentoflavones, as described in the literature, with analgesic, antibacterial, anti-inflammatory, antioxidant, and anti-tumor properties [47,49].

Other compounds identified in the genus *Garcinia* are 7-epiclusianone, with antiphylactic, anti-inflammatory, antiparasitic, trypanocide, antiproliferative, and cytotoxic activity, [50,51] and Garcinia fenone, with antiproliferative and antimicrobial activity [53].

Fruits and leaves may vary in relation to bioactive compounds and physical chemical composition due to several factors such as location, vegetation, degree of ripeness, processing, storage, and climatic conditions [16].

The fruits of *Garcinia gardneriana* showed a high moisture content (82.17%), as other species, such as *Garcinia brasiliense* (80.3 ± 1.70%). The moisture content of *Garcinia humilis* (87.30 ± 0.94%) and *Garcinia xanthochymus* (89.04 ± 0.13%) pointed out that several factors, such as season, climate, production period, and others, can influence the water content in fruits [11].

As for the protein content, the fruits presented 1.35 per 100 g^−1^, as expected for fruit pulps, corroborating with those who cited several native Cerrado fruits, such as araçá (*Psidium cattleyanum*) (0.50 g 100 g^−1^), araticum (*Annona crassiflora*) (1.22 g 100 g^−1^), murici (*Byrsonima crassifolia*) (0.72 g 100 g^−1^), and Brazilian Cerrado cashew (1.18 g 100 g^−1^), with lower values [51].

In the human body, phenolic compounds act by eliminating free radicals, promoting protection against oxidative stress, and generating additional health benefits. The quantification of the biological effects of phenolic compounds is important, as it becomes a way to evaluate the antioxidant properties of a plant species [52].

In this study, the value found on the leaf was greater than that described for the same species using the same methodology (28.06 and 9.22 mg GAE 100 g^−1^) for leaves and branches, respectively [55]. In leaves of *G. atrovirids* with average concentrations of 1.792 g gallic acid 100 g^−1^, 34.39 ± 0.48 g gallic acid 100 g^−1^ in aqueous extract of *G. brasiliensis* and 15.98 ± 0.79 g gallic acid 100 g^−1^ in ethanol extract was found [56,57].

On the other hand, higher values were found in studies carried out on the fruit pulp regarding the presence of phenolic compounds in *G. humilis* (373 mg GAE 100 g^−1^) and *G. xantochymus* (100.55 mg GAE 100 g^−1^) [11] and in fruits of *G. pedunculata* (189.8 mg ± 65.0 GAE 100 g^−1^) and *G. Morella* (183.0 mg ± 62.0 GAE 100 g^−1^) [17]. The leaf aqueous extract of *Garcinia brasiliensis* presented 343.98 ± 4.8 mg GA/g and Fukugiside as a major component and showed important antimicrobial and antioxidant properties, besides not having shown cytotoxic activity in human dermal fibroblasts, making its use a potential for the development of new drugs for cutaneous lesions’ treatment [12].

Vitamin C is as an important natural nutrient in the daily diet [27]. Fruits offer several health benefits and are part of a group of foods rich in bioactive compounds, mainly antioxidants [28]. The ascorbic acid reduces tocopherol radicals to their active forms on cell membranes providing anticancer potential [29].

The ascorbic acid content of *Garcinia gardneriana* showed lower values for fruits (25.23 mg 100 g^−1^) and higher for leaves (30.26 mg 100 g^−1^). However, they are considered higher when compared to other fruits of the same family and species: Bacuri (*Garcinia gardneriana*) (2.4 mg 100 g^−1^), *Garcinia cochinchinensis* Choisy (11.62 mg 100 g^−1^), and pulp of *Garcinia madruno* (24.74 mg 100 g^−1^) [1].

However, 40.32 mg 100 g^−1^ in the pulp of *Garcinia xanthochymus* Hook was reported, which is higher than that found in the present study [26].

The recommended daily intake (RDI) of vitamin C, according to current Brazilian legislation ANVISA (National Health Surveillance Agency), RDC (Resolution of the Collegiate Board)- 269/05 is 45 mg (recommendation for an adult). This corroborates studies that have shown that both the consumption of fruits and natural juices benefits the body for maintaining the balance of the antioxidant system and by improving plasma levels of vitamin C among other compounds of this nature [58,59].

Carotenoids are present mainly in vegetables and fruits, being efficient in organic protection against carcinogenesis. In vitro and in vivo tests with carotenoids have played important roles in the treatment and prevention of cancer, demonstrating an effective antioxidant action [30].

The contents of carotenoids in the pulp of fruits of the species *Garcinia brasiliensis* was 191.8 mg of β-carotene 100 g^−1^ [52]. However, it was reported that achachairu (*Garcinia humilis* Vahl) and yellow mangosteen (*Garcinia xanthochymus* Hook) had carotenoid contents of 61.4 and 134.82 mg of 100 g β-carotene^−1^, respectively. These values are higher than those found in the present study [60].

The fruit and the leaf can be considered as a rich source of carotenoids as they had a content greater than 20 μg/g^−1^ (2 mg/100 g^−1^), which is the minimum value considered for a food to be a source of this compound [57].

As for the antioxidant activity of extracts, the chloroform fraction of leaves showed a greater activity, i.e., a greater antioxidant action than DPPH, with a CI_50_ 14.27 μg/mL, as well as the ethyl acetate and ethanolic extract fractions, with CI_50_ 16.68 μg/mL and 16.95 μg/mL, respectively. Studies carried out on the hydroalcoholic extract of bacupari leaves obtained a CI_50_ of 34.83 μg/mL [31].

So far, no reports on whole fruits have been found, but only specific and isolated analyses such as skin, pulps, and seeds. In fruits, the hexane extract showed the lowest CI_50_ (20.05 μg/mL) compared to the other studied fractions. The antioxidant activity in other species of *Garcinia* was studiedand a CI_50_ 54 μg/mL for *Garcinia humilis* and 40.77 μg/mL for *Garcinia xantochymus* was verified [11].

Compared to the method of reducing capacity of the reagent Folin–Ciocalteau, the relations repeated: Leaves presented a higher phenolic concentration with a better result in relation to fruits. An important correlation between antioxidant activity and phenolic compounds has also been reported in other studies on fruits [60,61].

Antitumor biological activity exerted by melon residues can be explained by the presence of phenolic content, such as flavonoids and tannins, and the high antioxidant capacity demonstrated by in vitro assays. We observed antiproliferative effects for melon residues’ extracts on tumor cells that might have been caused by the activation of cell death mechanisms. Several antioxidant mechanisms of compounds present in melon peels and seeds might be associated with antiproliferative effects. Several enzymes, such as cytochrome C oxidase, ascorbate oxidase, and superoxide dismutase, are involved in cellular redox mechanisms [14].

Dietary antioxidants may be beneficial in preventing neoplasia due to their role in terms of immune response, since phagocytic cells produce free radicals as a defense of the body against the infection generated, and an adequate intake of these antioxidants is relevant to prevent damage caused by oxidant immune cells themselves [43].

Phenols’, flavonoids’, tannins’, and alkaloids’ contents were estimated much higher in polar fractions like fractionated ethyl acetate (TBEE), butanol (TBBE) and water (TBWE).

Thus, the presence of these important phytochemicals in the polar fractions might play an important role in establishing phytochemical content-function relationship, explaining their antioxidant and antiproliferative activity through various mechanisms like cell cycle arrest and apoptosis. Polar fractions of *Terminalia belerica* were seen to act as potent in vitro reactive oxygen and nitrogen species scavengers. Moreover butanol (TBBE) and water (TBWE) fractions were observed to be selectively cytotoxic towards breast cancer (MCF-7), cervical cancer (HeLa), and glioblastoma (U87) cell lines. These fractions arrested growth of MCF-7, HeLa, and U87 cells at G2/M phase, while ethyl acetate fraction (TBEE) caused apoptosis to check the growth of these cancer cells but none showed toxicity towards normal fibroblast cells (WI-38 cell line). These observations were further confirmed with imaging where arrested cells did not incorporate BrdUin DNA as compared to control cells. Moreover, butanol and water extracts of *Terminaliabelerica* upregulated p53 and p21 tumor suppressor proteins in MCF-7, HeLa, and U87 cells, which corroborated with G2/M arrest and apoptosis. Hence, our findings suggest *T. belerica* polar fractions (TBEE, TBBE, TBWE) as potent antioxidant and anticancer extracts which can be selectively used as a remedy against various types of cancer, probably due to the presence of various bioactive compounds identified [62].

The antioxidants do not always promote cell proliferation. Several studies report that antioxidants contribute to the anticancer effect, as long as their concentrations are evaluated. Apoptosis is cell death mechanisms induced by the intracellular increase in the generation of free radicals, thus causing the inhibition of tumor growth. Higher antioxidants’ concentrations decrease the generation of free radicals and cause inhibition of apoptosis. While normal concentrations of Glutathione (GSH) and other antioxidants in the intracellular space protect nuclear DNA from lesions caused by reactive oxygen species and decrease the prevalence of cancer. Normal amounts of Glutathione GSH and other antioxidants in the cancer already installed cause an increase in malignant cell proliferation and a decrease in apoptosis and facilitate the tumor neoangiogenesis [40,63].

Human antioxidative defense system includes superoxide dismutase (SOD), catalase (CAT), glutathione peroxidase (GPx), glutathione (GSH), and others, and allows the elimination of excess reactive oxygen species (ROS) including, among others, superoxide anions (O_2_^−^•), hydroxyl radicals (OH•), alkoxyl radicals (RO•), and peroxyradicals (ROO•). However, our endogenous antioxidant defense systems need exogenous antioxidants originating reducing compounds such as vitamin C, vitamin E, carotenoids, and polyphenols. There is continuous demand for exogenous antioxidants in order to prevent oxidative stress, representing a disequilibrium redox state in favor of oxidation [64].

Several studies show controversial results of exogenous antioxidants, discussing that the type, dosage, and matrix of exogenous antioxidants may be determining factors impacting the balance between beneficial or deleterious effects of these natural compounds. Double-edged effects of exogenous antioxidants on cellular responses include oxidative, nitrosative, and dicarbonyl metabolisms and other pathways such as inflammatory processes, depending potentially on their concentrations: Physiologic doses lead to beneficial effects, whereas high doses may result in harmful effects [64].

Different concentrations of antioxidants were reported to generate diverse biological effects, and higher or lower concentrations showed cytotoxicity in several cancer cell lines [65,66]. Other studies demonstrated a relationship between the antioxidant activity of extracts and, consequently, their anticancer activity in cancer cell lines, based on the calculated Pearson coefficient. Oftentimes, this correlation was not found between the anticancer and antioxidant activity [66,67].

Our results showed that the EEF and EEFH, with the values of GI_50_ lower than 30 µg/mL, possess the best antioxidant properties (IC_50_ 39.13 µg/mL and 20.20 µg/mL). However, EEFC did not possess free radical scavenging properties and was also one of the most active anticancer agents.

Its antiproliferation activity could be related to different mechanisms of action. Our data cannot prove if the presence of polyphenols are responsible for this biologic activity, even though the value was considerate moderate (100–500 mg/100 g^−1^ of GAE). It was the same for the ascorbic acid. Content showed values 25.23 mg/100 g^−1^ and 30.26 mg/100 g^−1^ in fruits and leaves. The vitamin C has antioxidant properties and protective action against breast cancer [68].

EEF and EEFH appear to be good sources of phytochemicals with antioxidant and potential anticancer activities. This study provides the initial evidence of the potential of these extracts as antiproliferative agents and their possible chemopreventive activity via their antioxidant properties.

## 4. Materials and Methods

### 4.1. Plant Material and Samples’ Preparation

*Garcinia gardneriana* fruits and leaves were collected in Campo Grande, Brazil (geographical coordinates: Latitude −20.533720 and longitude −54.675146) and identified by a botanist from Campo Grande, Mato Grosso do Sul (Herbarium of the Universidade Federal de Mato Grosso do Sul, where a voucher, specimen no. 54611, is deposited).

The dried fruits (354.84 g) and leaves (116.13 g) of *Garcinia gardneriana* were powdered separately and extracted with ethanol by maceration for seven days. The resulting solutions were filtered and concentrated under reduced pressure at maximum temperature of 38 °C, yielding 316.38 g of ethanolic extract of fruits and 116.13 g of ethanolic extract of leaves.

Aliquots of dried ethanol extracts of fruits (EEF 25.08 g) and leaves (EEL –042.00 g) were separately solubilized in 500 mL of methanol:water solution (9:1) and then fractionated by partition with hexane, chloroform, and ethyl acetate, resultin, in four fractions for each ethanolic extract: Hexane (EEFH 2.85 g and EELH 11.52 g), chloroform (EEFC 0.34g and EELC 12.84 g), ethyl acetate (EEFA 5.91 g and EELA 3.07 g), and hydromethanol (EEFW 14.08 and EELW 11.25 g). The eight fractions were concentrated under reduced pressure at 38 °C and kept under refrigeration with their respective extracts, totaling 10 samples.

### 4.2. Chemical Profile of EEFH and EELH

The extracts and fractions (EEF, EEFC, EEFA, EEFW, EEL, EELC, EELA, EELW, 5 mg/mL) were analyzed using a high-performance liquid chromatography (Prominence UFLC, Shimadzu, Kyoto, Japan) coupled to a diode-array detector (190–400 nm) and a mass spectrometer with an electrospray ionization (ESI) source and the quadrupole-time-of-flight (QTOF) (MicrOTOF-Q III, Bruker Daltonics, Billerica, MA, USA) analyzers operating in negative ion modes. Nitrogen was used as gas of nebulization (4 bar), dry (9.1 L/min at 200 °C) and collision. The capillary voltage was set at 3500 V and the scan range was *m*/*z* 100–1300. The analysis was performed on a C-18 column (Kinetex, 150 mm × 2.1 mm id, 2.6 μm) with an oven temperature of 40 °C. The mobile phase was deionized water (A) and acetonitrile (B), both containing 0.1% formic acid (*v*/*v*), under the following gradient profile: 0–2 min 3% B, 2–25 min 3–25% B, and 25–40 min 25–80% B. The flow rate was 0.3 mL/min and the injection volume was 5 μL. The extracts were prepared at 1 mg/mL using acetonitrile and water (85:15, *v*/*v*) and filtered on a 0.22 μm × 3.0 mm PTFE (Polytetrafluoroethylene) membrane (Millex^®^, Millipore, Sigma-Aldrich, St. Louis, MO, USA).

### 4.3. Centesimal Composition of Fruits and Leaves of Garcinia gardneriana

The analyses were performed in triplicate on fruits and leaves of bacupari. The following parameters were determined: Moisture (determined in an oven at 105 °C until constant weight [30]) analyzed in a muffle furnace at 550 °C, lipids (determined by direct extraction with organic solvent in a Soxhlet apparatus), and proteins (determined by the classic micro-Kjeldahl method using a nitrogen into protein conversion factor of 5.75) [69]. The determination of carbohydrates (including fiber) was performed by theoretical calculation (difference) in the results of the triplicates according to the formula:

*%Carbohydrates = 100 − (%moisture + %proteins + %lipids + %ash)*. (1)

### 4.4. In Vitro Cytotoxicity Assay

The 10 samples were tested against the following tumor cell lines from the American Type Culture Collection (ATCC, Manassas, VA, USA): Murine melanoma (B16-F10), human breast adenocarcinoma (MCF-7), kidney adenocarcinoma (786-0), and colon carcinoma (HT-29). All of them were donated by Prof. Dr. João Ernesto de Carvalho (Center for Chemical, Biological and Agricultural Studies-CPQBA-Unicamp, Brazil). They were also tested on murine fibroblast (NIH-3T3) cell line obtained from the Cell Bank of Rio de Janeiro (Rio de Janeiro, Brazil).

The B16-F10 and NIH-3T3 cells were grown in high-glucose DMEM (Dulbecco’s modified GAE medium) and the other lines in RPMI-1640 (Roswell Park Memorial Institute Medium) (Sigma-Aldrich, St. Louis, MO, USA), both containing 10% fetal bovine serum (Gibco, Thermo Fisher Scientific, Waltham, MA, USA), streptomycin (100 µg/mL), and penicillin (100 U/mL) (Sigma-Aldrich, St. Louis, MO, USA). The cells were subsequently distributed in 96-well plates (100 μL/well) and exposed for 48 h to increasing concentrations of extracts and their respective fractions (0.25, 2.5, 25, and 250 μg/mL) (log_10_ scale). Dilutions were prepared inDimethylsulfoxideDMSO (0.1%). Cell proliferation was determined using the colorimetric method with sulforhodamine B (SRB) (Sigma, St. Louis, MO, USA). Using the concentration-response curve for cell lines, the GI_50_ (concentration causing 50% cell growth expressed in μg/mL) was determined by nonlinear regression analysis (sigmoidal fitting) using the software Origin 6.0 (OriginLab Corporation, Northampton, MA, USA) [12].

According to the National Cancer Institute (NCI), the extract with GI_50_ < 30 μg/mL will be considered active [70]. In this study, GI_50_ values < 250 μg/mL were considered active and GI_50_ > 250 μg/mL were inactive.

### 4.5. Antioxidant Property

#### 4.5.1. Determination of Total Phenols

The quantification of phenolic compounds was performed using the Folin–Ciocalteau method. The ethanolic extracts were dissolved in methanol to obtain a concentration of 0.5 mg/solids/mL^−1^ and then analyzed. The total amount of phenols in each extract was quantified using a standard curve prepared with gallic acid and expressed as gallic acid equivalent (GAE) [37].

#### 4.5.2. Evaluation of Antiradical Activity (DPPH)

The method used to determine the antioxidant capacity in food extracts uses the reagent 2,2-difenil-1-picrilhidrazil (DPPH). The stable radical DPPH was used to assess the antiradical activity of natural antioxidants by assessing their ability to sequester free radicals. It was estimated according to the method adapted from Brand-Williams (1995) [18] using the microdilution technique in 96-well microplates. The radical scavenging activity was evaluated based on the 50% inhibition values (IC_50_), which correspond to the amount of sample needed to inhibit the 50% DPPH oxidation. Data on the percentage of DPPH inhibition were obtained graphically from the absorbance values and log 10 of the concentrations. The values were determined by linear regression (Y = a + bx), such as “IC_50_ = (50 + b)/a”, subsequently adjusted to a power of 10 and expressed in μg/mL of solution.

#### 4.5.3. Determination of Ascorbic Acid and Carotenoids

The determination of ascorbic acid content was performed by titration with the reagent 2,6-dichlorophenolindophenol. The reading was taken in triplicate using a Libra Biochrom S60PC spectrophotometer at 450 nm and petroleum ether as blank. After the reading, the calculation of the carotenoid content was determined [18].

### 4.6. Statistical Analyses

All results were expressed as mean ± standard error of the analyzed triplicate. The difference between groups was determined by one-way ANOVA (GraphPad Prism 5^®^, San Diego, CA, USA). Values were considered significant when *p* < 0.05.

## 5. Conclusions

*Garcinia* is a potential source of bioactive compounds with a significant antiproliferative effect in breast neoplastic lines (MCF), this being the second type of cancer with the highest incidence in women. In addition, the best antioxidant activity of *Garcinia* was found in the hexane fraction of fruits and in the chloroform fraction of leaves. Both fruits and leaves have a high vitamin C content, exceeding by more than 50% the recommended daily intake (RDI). It can be consumed fruit or as juice and tea and other preparations, since 100 g of fruits contain 56.06% and leaves contain 67.24% of the recommended daily dose. Fruits have a lower protein and carbohydrate content than leaves, but a higher amount of lipids. Leaves indicate a high content of proteins, carbohydrates, and ashes (7.64%, 54.79%, and 4.93%, respectively). Due to the high ash content found in leaves, it is relevant to evaluate the mineral profile. EEF and EEFH appear to be good sources of phytochemicals with antioxidant and potential anticancer activities. Further and more detailed studies should be carried out to understand the mechanism of antiproliferative activity against cancer cells lines.

## Figures and Tables

**Table 1:** Compounds tentatively identified from the ethanol extract of *Garcinia gardneriana* fruits (EEF).

Substance	Rt (min)	Compound	[M-H]^−^ (MF)	Precursor ion (*m/z*)	Fragment Ions (*m/z*)	UV
**1**	1.1	Dimers of hexoses	C_12_H_21_O_11_	341.1097		
**2**	1.1	Monomers of hexoses	C_6_H_11_O_6_	179.0551		
**3**	1.2	Hydroxycitric acid lactone	C_6_H_5_O_7_	189.0034		
**4**	1.3	Glycosylated citric acid	C_12_H_17_O_12_	353.0725		
**5**	1.4	Citric acid	C_6_H_7_O_7_	191.0191		
**6**	29.4	GB-2a biflavonoid	C_30_H_21_O_11_	557.1105		280
**7**	30.0	Fukugetine	C_30_H_19_O_11_	555.0935	429, 403, 401, 295	280
**8**	31.3	Volkensiflavone	C_30_H_19_O_10_	539.0982	413, 387	280

Note: MF, molecular formula; RT, retention time; UV, ultraviolet. All MFs were determined from the accurate mass considering a mass error and mSigma lower than 8 ppm and 30, respectively.

**Table 2:** Compounds tentatively identified from the ethanol extract of *Garcinia gardneriana* leaves (EEL).

Substance	Rt (min)	Compound	[M-H]^−^ (MF)	Precursor Ion (*m/z*)	Fragment Ions (*m/z*)	UV
**1**	1.2	Dimers of hexoses	C_12_H_21_O_11_	341.1097		
**2**	1.2	Monomers of hexoses	C_6_H_11_O_6_	179.0551		
**3**	1.2	Hydroxycitric acid lactone	C_6_H_5_O_7_	189.0052		
**6**	29.4	GB-2a biflavonoid	C_30_H_21_O_11_	557.1105	431, 295	280
**7**	30.0	Fukugetine	C_30_H_19_O_11_	555.0956	429, 403, 401, 295	280
**8**	31.3	Volkensiflavone	C_30_H_19_O_10_	539.0982	413, 387	280
**9**	1.2	Tetramer of hexoses	C_24_H_43_O_22_	683.2259	341, 179	
**10**	12.1	Procyanidin B	C_30_H_25_O_12_	577.1361	407, 289	280
**11**	12.5	epicatechin	C_15_H_13_O_6_	289.0724		280
**12**	17.9	Vitexin	C_21_H_19_O_10_	431.1000	311, 283	273, 330
**13**	18.2	Vitexin-O-rhamnoside	C_27_H_29_O_14_	577.1564	413, 293	270, 340
**14**	18.6	Isovitexin	C_21_H_19_O_10_	431.0996		273, 330
**15**	23.0	Tetrahydroxy-xanthone	C_13_H_7_O_6_	259.0251	215, 187	275, 315, 360
**16**	24.0	GB-2a-I-7-O-glucoside	C_36_H_31_O_16_	719.1597	431, 313, 295	284
**17**	24.5	Fukugiside	C_36_H_29_O_16_	717.1501	565, 493, 429, 403	276, 350
**18**	32.0	Amentoflavone	C_30_H_17_O_10_	537.0834	443, 417, 399, 375, 331	269, 336
**19**	33.7	Prenylated Xanthone	C_18_H_15_O_6_	32.0877	311, 295, 272	275, 316
**20**	34.0	Prenylated Xanthone	C_18_H_15_O_6_	327.0888	283, 272, 258, 243	275, 320
**21**	34.4	7-O-methylamentoflavone	C_31_H_19_O_10_	551.0995	457, 431, 413, 389, 345	272, 330
**22**	34.5	Amentoflavone	C_35_H_25_O_11_	621.1426	551, 441, 431, 389, 345	276, 328
**23**	35.4	Prenylated Xanthone	C_18_H_15_O_8_	311.0920	295 283, 267, 255	277, 307
**24**	36.2	Garciniaflavone D	C_35_H_25_O_10_	605.1458	511, 485, 467, 443, 399, 374, 309, 227	275, 333
**25**	42.8	7-epiclusianone	C_33_H_41_O_4_	501.3007	417, 347, 305, 175	276, 307
**26**	43.1	Garciniaphenone	C_28_H_33_O_4_	433.2390	349, 295, 279, 241	278, 307

Note: MF, molecular formula; RT, retention time; UV, ultraviolet. All MFs were determined from the accurate mass considering a mass error and mSigma lower than 8 ppm and 30, respectively.

**Table 3:** Centesimal composition and bioactive compounds of fruits and leaves of *Garcinia gardneriana*.

Parameter	Fruit Mean ± MSE	Leaf Mean ± MSE
Moisture (%)	82.17 ± 0.91 ^a^	30.51 ± 2.77 ^b^
Ash (%)	0.40 ± 0.015 ^a^	4.93 ± 0.06 ^b^
Protein (%)	1.35 ± 0.12 ^a^	7.4 ± 0.08 ^b^
Lipids (%)	5.41 ± 0.1^a^	2.11 ± 0.09^b^
Carbohydrates (%) **	10.64 ± 0.83 ^a^	54.79 ± 2.89 ^b^
Phenols (mg GAE 100 g^−1^)	107.07 ± 9.65 ^a^	132.46 ± 2.32 ^a^
Vit C (mg 100 g^−1^)	25.23 ± 3.58 ^a^	30.26 ± 2.01 ^a^
Carotenoids (mg β-carotene 100 g^−1^)	27.05 ± 4.04 ^a^	76.25 ± 3.51 ^b^

Different letters on rows indicate significant difference by Tukey test (*p* < 0.01); MSE, mean standard error; mean value ± standard deviation; n = 3. ** Calculations by difference. GAE: Gallic acid equivalent.

**Table 4:** Determination of antioxidant activity in vitro, CI_50_ (50% inhibition values)data (µg/mL), by the DPPH (radical scavenging activity using DPPH (1,1-diphenyl-2-picrylhydrazyl) assay) method for extracts and fractions of fruits (peel, pulp, and seed) and leaves of *Garcinia gardneriana*.

Extract and Fraction	Fruit (µg/mL)	Leaf (µg/mL)
Ethanol extract	39.13 ± 0.09 ^a^	16.95 ± 0.80 ^b^
Hexane fraction	20.20 ± 1.21 ^a^	27.03 ± 1.54 ^b^
Chloroform fraction	103.37 ± 3.32 ^a^	14.27 ± 1.36 ^b^
Ethyl acetate fraction	73.40 ± 10.72 ^a^	16.68 ± 0.63 ^b^
Hydromethanol fraction	166.64 ± 2.70 ^a^	35.46 ± 1.70 ^b^

Mean value ± standard deviation, n = 3. Different letters indicate significant difference by Tukey test.

**Table 5:** Cytotoxic activity, GI_50_ values* (μg/mL), for extracts and fractions of fruits and leaves of *Garcinia gardneriana*, lines 786 (kidney carcinoma), HT-29 (colon carcinoma), MCF-7 (breast carcinoma), B16-F10 (murine melanoma), and NIH/3T3 (murine fibroblast).

Line	Sample ***										
	EEF	EEFH	EEFC	EEFA	EEFHM	EEL	EELH	EELC	EELA	EELHM	Doxorubicin **
**786**	26.36	20.63	23.17	>250	>250	>250	>250	>250	>250	>250	0.026
**HT-29**	25.09	24.55	20.04	>250	>250	79.89	31.00	>250	>250	>250	0.24
**MCF-7**	21.42	15.81	17.22	>250	>250	50.29	28.7	36.29	>250	>250	0.025
**B16F10**	24.71	23.43	24.67	>250	>250	60.92	33.79	80.59	>250	>250	0.026
**3T3**	25.32	24.63	27.10	>250	>250	39.87	>250	46.03	>250	>250	0.36

* Concentration that inhibits 50% of cell growth determined by nonlinear regression analysis using the software ORIGIN 6.0. ** Doxorubicin: Positive control. *** EEF (fruit ethanolic extract), EEFH (hexane fraction), EEFC (chloroform fraction), EEFA (ethyl acetate fraction), EEFW (hydromethanolic fraction), EEL (ethanol leaf extract), EELH (hexane fraction), EELC (chloroform fraction), EELA (ethyl acetate fraction), and EELW (hydromethanolic fraction).

**Table 6:** Biological activities of compounds identified in the extracts of *G. gardneriana* leaves and fruits. Modes and their biological activities were already reported in the references cited for the species *Garcinia*.

Classification	Compound	Activity	Reference
**Xanthones**	Tetrahydroxy-xanthone Prenylated xanthone	Anti-inflammatory, antiviral (herpes), antimicrobial, antifungal, cytotoxic, and antioxidant.	[34,38,44,45]
	epicatechin	Antioxidant	[34,40,41]
**Biflavonoids**	GB-2a biflavonoid	Anti-inflammatory, analgesic, antiviral, antioxidant activity	[11,46,47]
	Procyanidin B2	Anti-inflammatory	[11,46]
	Fukugetine	Anti-inflammatory, antibacterial	[11,32,46,48]
	Volkensiflavone	Analgesic, anti-tumor, antibacterial	[8,11,41]
	GB-2a-I-7-O-glucoside	Antibacterial	[32]
	Fukugisid	Analgesic	[11]
	7-O-methylamentoflavone		[44]
	Amentoflavone	Analgesic, antibacterial, antifungal, anti-inflammatory, contraceptive, antioxidant, antitumor, antiviral, and cytotoxicity	[45,49]
**Benzophenones**	7-epiclusianone	Antimicrobial, high concentrations (vasoconstrictor action)/low concentrations (vasodilator), anti-caries, anti-anaphylaxis anti-inflammatory, antiparasitic, trypanocide, antiproliferative, cytotoxic	[50,51]
	Garciniaphenone	Antimicrobial, antiproliferative	[52]

## Abbreviation List:

EEF	Fruit ethanolic extract
EEFA	Ethyl acetate fraction
EEFC	Chloroform fraction
EEFH	Hexane fraction
EEFW	Hydromethanolic fraction
EEL	Ethanol leaf extract
EELA	Ethyl acetate fraction
EELC	Chloroform fraction
EELH	Hexane fraction
EELW	Hydromethanolic fraction
MF	Molecular formula
MS/MS	Tandem mass spectrometry
Ppm	Parts per million
Rt	Retention time
[M-H]	Deprotonated ion
m/z	mass-to-charge ratio
